# Supplementary material for: DMRforPairs: identifying Differentially Methylated Regions between unique samples using array based methylation profiles
Source: BMC Bioinformatics. 2014 May 15;15:141. doi: 10.1186/1471-2105-15-141 (PMC4046028; doi:10.1186/1471-2105-15-141)
Supplement: Additional file 2 — DMRforPairs output for the comparison of A431-MCF7 and NA17018-NA17105. Please start from the HTML files in each folder. Available via the BMC Bioinformatics website. [file 1471-2105-15-141-S2.zip › 1394847754114233_MOESM2_ESM/A431_MCF7/figures/40673.pdf]

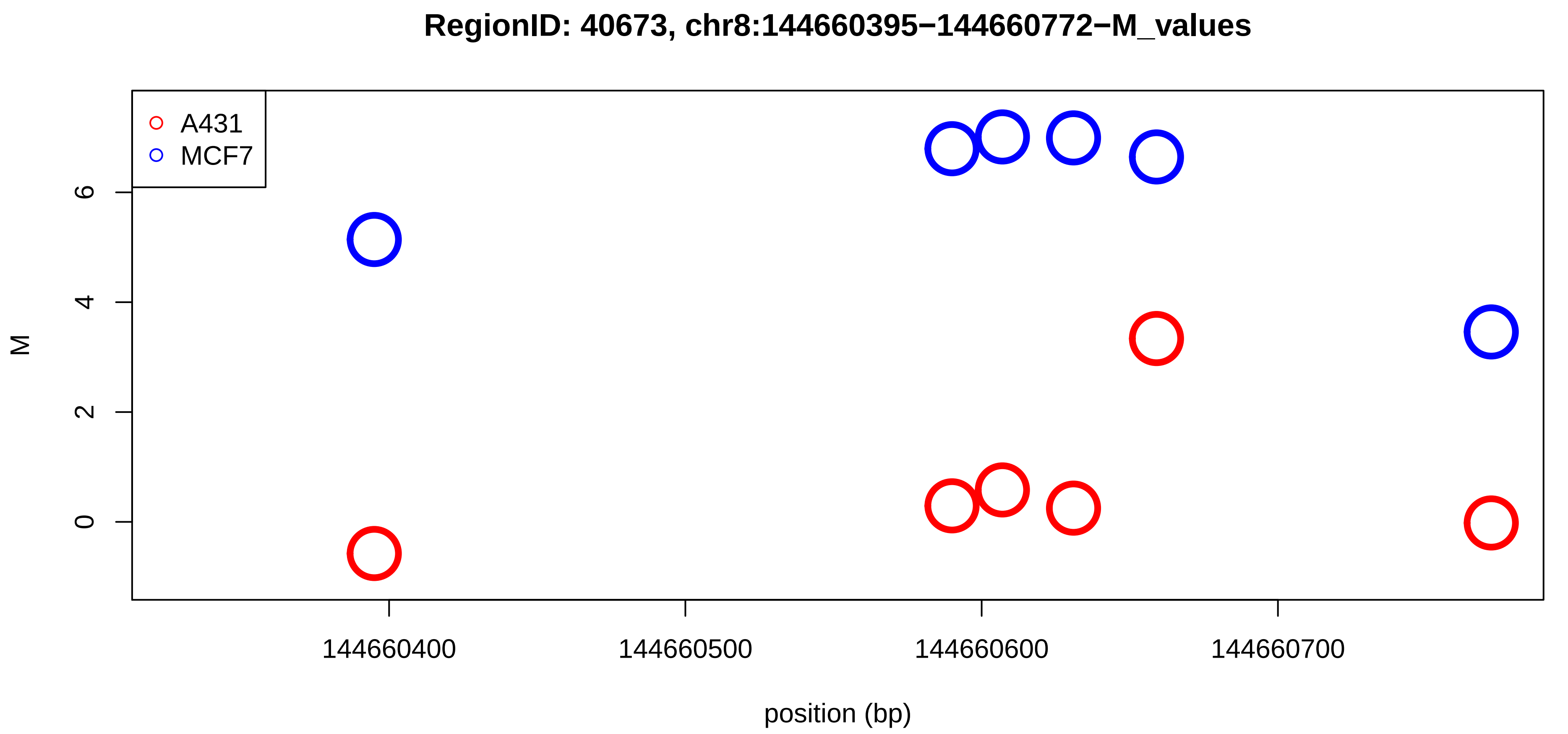
RegionID: 40673, chr8:144660395−144660772−M_values
A431
MCF7
M
0
2
4
6
144660400
144660500
144660600
144660700
position (bp)

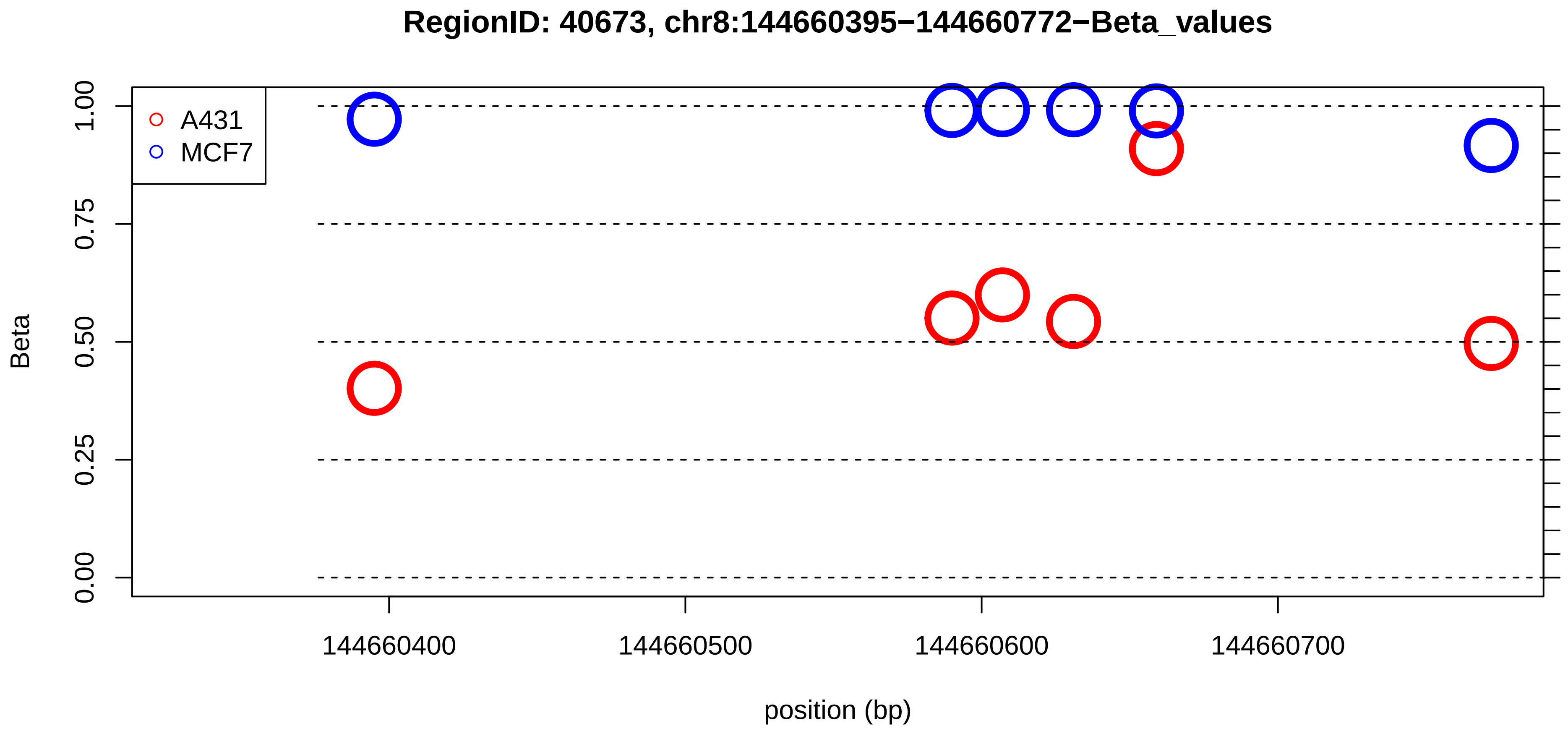
RegionID: 40673, chr8:144660395−144660772−Beta_values
A431
MCF7
Beta
0.00
0.25
0.50
0.75
1.00
144660400
144660500
144660600
144660700
position (bp)

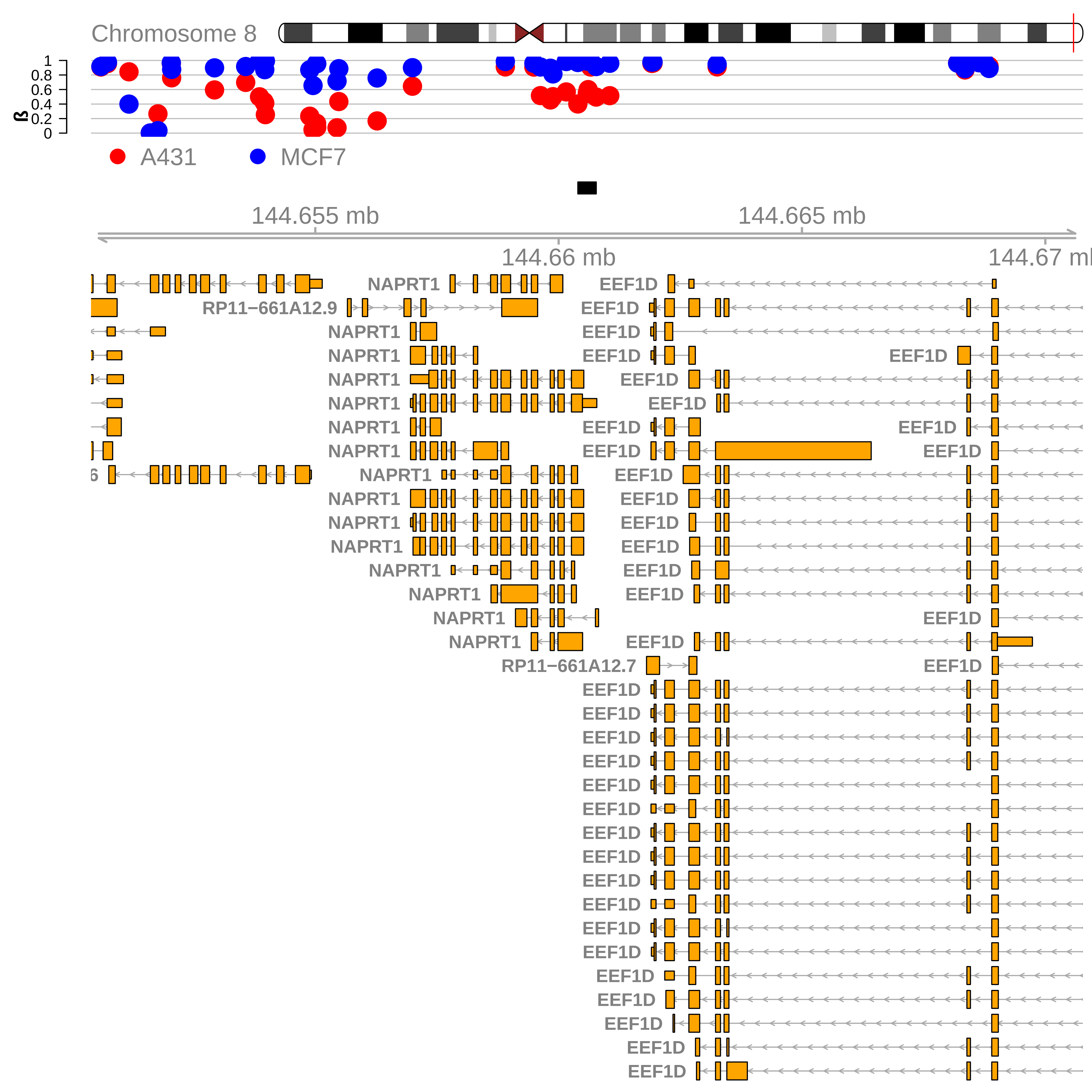

Chromosome 8
β
1
0.8
0.6
0.4
0.2
0
A431
MCF7
144.655 mb
144.665 mb
144.66 mb
144.67 mb
NAPRT1
EEF1D
RP11−661A12.9
EEF1D
NAPRT1
EEF1D
NAPRT1
EEF1D
EEF1D
NAPRT1
EEF1D
NAPRT1
EEF1D
NAPRT1
EEF1D
EEF1D
NAPRT1
EEF1D
EEF1D
NAPRT1
EEF1D
NAPRT1
EEF1D
NAPRT1
EEF1D
NAPRT1
EEF1D
NAPRT1
EEF1D
NAPRT1
EEF1D
NAPRT1
EEF1D
NAPRT1
EEF1D
RP11−661A12.7
EEF1D
EEF1D
EEF1D
EEF1D
EEF1D
EEF1D
EEF1D
EEF1D
EEF1D
EEF1D
EEF1D
EEF1D
EEF1D
EEF1D
EEF1D
EEF1D
EEF1D
EEF1D
